# Optimal Pressure Sensor Deployment for Leak Identification in Water Distribution Networks

**DOI:** 10.3390/s23125691

**Published:** 2023-06-18

**Authors:** Guang Yang, Hai Wang

**Affiliations:** School of Mechanical Engineering, Tongji University, No. 1239, Siping Road, Shanghai 200092, China; 2010346@tongji.edu.cn

**Keywords:** leak identification, pressure sensor deployment, water distribution networks

## Abstract

Pipe leakage is an inevitable phenomenon in water distribution networks (WDNs), leading to energy waste and economic damage. Leakage events can be reflected quickly by pressure values, and the deployment of pressure sensors is significant for minimizing the leakage ratio of WDNs. Concerning the restriction of realistic factors, including project budgets, available sensor installation locations, and sensor fault uncertainties, a practical methodology is proposed in this paper to optimize pressure sensor deployment for leak identification in terms of these realistic issues. Two indexes are utilized to evaluate the leak identification ability, that is, detection coverage rate (*DCR*) and total detection sensitivity (*TDS*), and the principle is to determine priority to ensure an optimal *DCR* and retain the largest *TDS* with an identical *DCR*. Leakage events are generated by a model simulation and the essential sensors for maintaining the *DCR* are obtained by subtraction. In the event of a surplus budget, and if we suppose the partial sensors have failed, then we can determine the supplementary sensors that can best complement the lost leak identification ability. Moreover, a typical WDN Net3 is employed to show the specific process, and the result shows that the methodology is largely appropriate for real projects.

## 1. Introduction

A water supply network is an important element of civil infrastructure for meeting the needs for people’s production and living water. Due to aging, corrosion, and human impacts on pipelines, pipeline leakage accidents occur frequently and result in a series of economic and social problems, such as water pollution and land subsidence [1]. According to the *Statistical Yearbook of Urban Water Distribution Networks* of China, the average leakage rate, which refers to the proportion of lost water volume to the total water volume entering the distribution network, decreased from 14.57% in 2017 to 12.68% in 2021 [2]. However, there is still a gap compared to developed European countries. The average leakage rates of most countries remain below 10% and even below 5% in Germany and the Netherlands [3]. China’s Ministry of Housing and Urban–Rural Development and the National Development and Reform Commission (NDRC) jointly issued a notice for strengthening the leakage control of public water supply pipe networks, which requires that the leakage rates of urban public water supply pipe networks nationwide should be controlled to less than 9% by 2025. With the developments in science and society, fine management requirements for the water supply industry have been proposed, and the leakage rates are expected to be further decreased to bring more social and economic benefits, with the exception of the District Metering Area (DMA) management, which deducts the metering losses [4].

Nowadays, the methods for leak identification can be roughly divided into three categories: equipment-based methods, transient-based methods, and data-driven methods [5,6]. The first methods relay information using hardware devices installed in the pipelines, and when leakage occurs, the devices can detect and determine the specific locations of the leaks. The representative equipment-based methods include sound-wave detection [7], the smart ball method [8], fiber optic cables [9], thermal imaging technology [10], etc. With Internet-of-Things (IoT) technologies [11], the transient-based methods and data-driven methods have been popular methods for leak location identification in practical engineering. The transient-based methods [12] and data-driven methods [13] rely on real-time monitoring hydraulic data, with the latter being more seriously dependent on these data. The difference is that the transient-based methods have a fundamental physical model while the data-driven methods mainly utilize various machine learning algorithms, such as the genetic algorithm [14], support vector machine [15], and neural networks [16,17], to predict pressures/flows compared with observed values [18]. Hydraulic data in water supply network objectively reflect the operating status of an entire network and provide the basis for various leak identification methods. Pressure sensors have the advantages of being low cost and non-invasive, with rapid response capabilities [19]. However, the overuse of pressure sensors will increase the cost burden and consume redundant manpower and material resources. Therefore, optimizing the pressure sensor arrangement in a water supply network is essential for improving operations management on the promise of a limited budget.

Compared to the studies on leakage detection and location, there are few researchers who have dealt with pressure sensor layout optimization focused on leak identification. Generally, the limited studies on optimal pressure sensor deployment in WDNs can be classified into those that used the sensitivity analysis method [20,21], the cluster analysis method [22], the correlation analysis method [23], and the optimization algorithm [24]. The optimization algorithm has the advantage of systematicity and reliability. However, the position of sensors in a large pipeline network is an explosive combination problem, and a more efficient and reasonable optimization method is necessitated to address this challenge. Juan Li et al. (2019) [25] proposed a novel semi-supervised strategy to optimize the sensor deployment that considered that some leak locations are unknown, and the results showed that the addition of the fuzzy c-means clustering method integrated with the original semi-joint mutual information (JMI) algorithm could efficiently improve the accuracy and stability of leak localization. Mengke Zhao et al. (2020) [26] proposed a net cost indicator based on the single-objective optimization and cost–benefit analysis models to determine an optimal set of sensor locations that would maximize the detection coverage rate under a fixed number of sensors. The method was demonstrated to be effective on the benchmark network Net3, and the sensor accuracy and pipe burst flow magnitude were shown to be key uncertainties. Narges Taravatrooy et al. (2020) [27] introduced a novel hybrid entropy-clustering approach where the redundant information from pressure sensors was reduced based on an information theory after the potential pressure sensors in WDNs were classified using a K-means clustering algorithm. Furthermore, the uncertainty of the pressure sensors’ error thresholds was analyzed by a set of fuzzy members using a triangular membership. These studies have all been based on multiple leakage events, and they separately considered some practical factors. However, there is little research that comprehensively contains realistic uncertainties that can be efficiently unitized in reality. Moreover, the leakage events in the representative studies mentioned above were all generated by EPANET [28,29]—famous water system simulation software—in which a burst pipe is simulated by adding an extra demand at a target node [30].

This paper puts forward a methodology for pressure sensor layout optimization with realistic issues and considers economic budgets (reflected by sensor accuracies and sensor amounts), available sensor installation locations, sensor faults, water demand profiles, etc. The smallest number of pressure sensors and the layouts can firstly be gained via sequential subtraction, and the abundant sensors can then be added to compensate for the lost information caused by sensor faults on the promise of an economic budget. Furthermore, the hydraulic model [31,32,33] utilized in this paper to generate a large number of leak samples is a novel method for simulating leakage incidents and can be set at any location in WDNs rather than at only nodes. The method is applied to a representative Net3 and the results show that it has an outstanding performance with respect to applicability and practicality.

## 2. Mathematical Model and Numerical Solution

In order to accurately analyze the leakage behavior of a pipeline, it is necessary to establish an advanced hydraulic model. A hydraulic model can comprise two aspects: a straight pipe or a pipe network.

### 2.1. Straight Pipe with Leakage

For analyzing the hydraulic behavior of water in a straight pipe, there are already several conventional models available. However, few of them consider water leakage in the middle of a pipe. In this paper, a mathematical model is introduced which can consider the influence of multiple leakage points.

**Figure 1:**
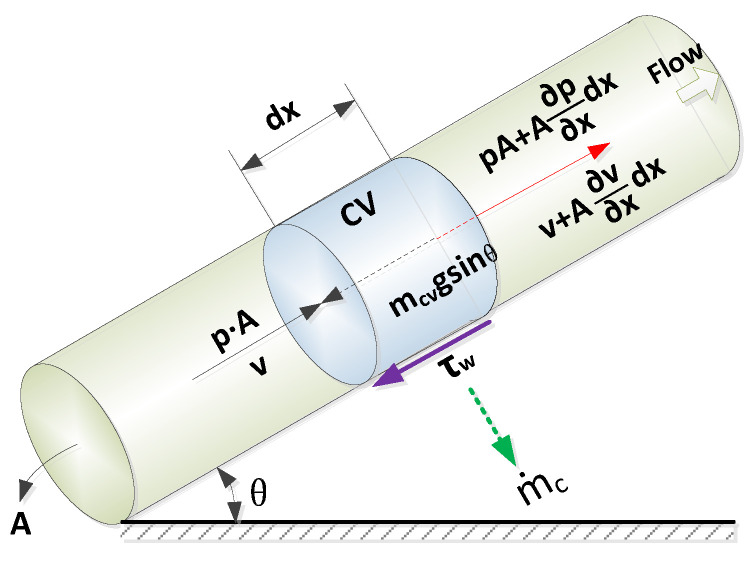
Water flow in a pipe.

**Figure 2:**
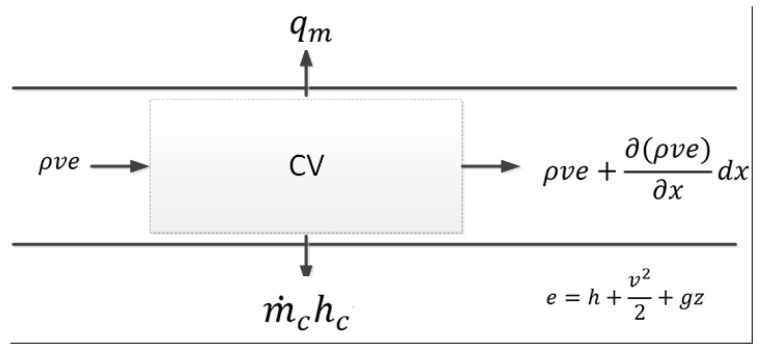
Energy into and out of the control volume.

The diameter of a pipeline is negligible compared to its length, which is generally hundreds to thousands of meters. Hence, only axial-direction water flow is considered in the proposed model. A control volume in a straight pipe (with a dip angle ± θ) with a length of dx was selected, shown in Figure 1. Time-dependent ordinary differential equations are derived from the mass and energy balances for each control volume, as shown in Figure 2. Taking the leakage loss out of a pipe wall, the mass conservation equations can be written as follows:

(1) $$\frac{\partial \rho }{\partial t}+\frac{\partial \left(\rho v\right)}{\partial x}={\dot{m}}_{c}$$

where ρ indicates the density of the water (kg/m^3^), v indicates the velocity (m/s), t indicates the time (s), x indicates the length of the pipeline (m), and $\dot{m_{c}}$ indicates the specific mass loss of the water leakage (kg/(m^3^·s)).

The momentum conservation equation can be written as

(2) $$\frac{\partial \left(\rho v\right)}{\partial t}+\frac{\partial \left(\rho v^{2}\right)}{\partial x}=-\frac{\partial p}{\partial x}-\frac{f\rho }{2d}v\left|v\right|-\rho gsin\theta$$

The energy conservation equation can be written as

(3) $$\frac{\partial }{\partial t}\left[\rho \left(u+\frac{v^{2}}{2}+gz\right)\right]+\frac{\partial }{\partial x}\left[\rho v\left(h+\frac{v^{2}}{2}+gz\right)\right]={\dot{m}}_{c}\cdot h_{c}+q_{m}$$

where p indicates the pressure (pa), u indicates the internal energy of the water (J/kg), d indicates the inner diameter of the pipeline (m), g indicates the gravity acceleration (m/s^2^), z indicates the relative height (m), h indicates the enthalpy value of the water (J/kg), $q_{m}$ indicates the specific heat loss from the water to the surrounding soil (W/m^3^), and $h_{c}$ indicates the enthalpy value of the water leakage (J/kg).

The state equation (Equation (4)) and the enthalpy equation (Equation (5)) for the water flow can be written as

(4) ρ=ρ(p,T)

(5) h=h(p,T)

Deductions about the mass, momentum, and energy conservation equations have been described many times in the literature. In this paper, a mathematical model is presented with additional water leakage modifications based on a single-phase flow simulator.

Consolidating Equations (1)–(3) into a matrix form, we can obtain

(6) $$\frac{\partial }{\partial t}\left[\begin{array}{c} \rho \\ \rho v\\ \rho \left(u+\frac{v^{2}}{2}+gz\right) \end{array}\right]+\frac{\partial }{\partial x}\left[\begin{array}{c} \rho v\\ \rho v^{2}+p\\ \rho v\left(h+\frac{v^{2}}{2}+gz\right) \end{array}\right]=\left[\begin{array}{c} {\dot{m}}_{c}\\ -\frac{f\rho }{2d}v\left|v\right|-\rho gsin\theta \\ {\dot{m}}_{c}\cdot h_{c}+q_{m} \end{array}\right]$$

To summarize, the thermal–hydraulic model presented in this paper is more appliable than other models when dealing with practical conditions. There are two main improvements: (1) the material loss caused by the leakage added to the pipeline mass conservation equation; and (2) the enthalpy value of the leaked water added to the energy conservation equation.

For sensor location analysis in a water pipe network, numerical simulations of steady-state flows are sufficient due to the ultrafast wave speed of water. Therefore, the time term in Equation (6) can be omitted for solving the discretized governing equations. In this way, Equation (6) can be simplified as follows:

(7) $$\frac{\partial }{\partial x}\left[\begin{array}{c} \rho v\\ \rho v^{2}+p\\ \rho v\left(h+\frac{v^{2}}{2}+gz\right) \end{array}\right]=\left[\begin{array}{c} {\dot{m}}_{c}\\ -\frac{f\rho }{2d}v\left|v\right|-\rho gsin\theta \\ {\dot{m}}_{c}\cdot h_{c}+q_{m} \end{array}\right]$$

There have been a number of developed methods that derive the discretized forms of mathematics equations. Here, the finite volume method (FVM) based on the QUICK scheme is adopted to perform spatial discretization. The pressure implicit split operator (PISO) is used to solve the discrete equation. The derivation method has been detailed in a previous study [31]. Regarding this paper’s focus on the WDNs, the energy conservation equation can be neglected during the numerical solution process.

### 2.2. Pipe Network

On the basis of the graph theory, the hydraulic regime of a pipe network can be described well. According to Kirchhoff’s current and voltage laws, a pipe network’s branch flow, pressure drop, and hydraulic resistance can be drawn using an analogy with the branch current, voltage, and electric resistance. In the network topology, *k* is the branch number and (*n* + 1) is the node number. The associated matrix *A*, an *n* × *k* order matrix, and the basic circuit matrix *B*, a (*k* − *n*) × *k* order matrix, of the pipe network can be gained. In the light of Kirchhoff’s current law, the equation can be expressed as

(8) $$A\cdot {\dot{V}}_{b}+P=0$$

where ${\dot{V}}_{b}$ is the flow rate column vector of each branch in the pipe network ($\left[{\dot{V}}_{b,1},{\dot{V}}_{b,2},\cdots ,{\dot{V}}_{b,k}\right]$), P is the flow rate column vector of each node ($\left[P_{1},P_{2},\cdots ,P_{n}\right]$).

From Kirchhoff’s voltage law, the following can be obtained:

(9) $$B\cdot \left(R_{b}-\rho gH_{p}\right)=0$$

where $R_{b}$ is the resistance column vector of each branch in the pipe network (${\left[R_{b,1},R_{b,2},\cdots ,R_{b,k}\right]}^{T}$), $H_{p}$ is the pump head column vector of each branch in the pipe network (${\left[H_{p,1},H_{p,2},\cdots ,H_{p,k}\right]}^{T}$), and the term in $H_{p}$ is zero unless there is a pump installed in the corresponding branch.

The resistance of the pipe branch k can be estimated by the Darcy–Weisbach equation as follows:

(10) $$R_{b,k}=f_{k}\cdot \frac{8\rho L_{k}{\left({\dot{V}}_{b,k}\right)}^{2}}{\pi^{2}{\left(d_{k}\right)}^{5}}$$

where $L_{k}$ is the length of the pipe branch k (m), $d_{k}$ is the inner diameter of the pipe branch k (m), and the friction factor $f_{k}$ is estimated by the Colebrook–White equation as follows:

(11) $$\frac{1}{\sqrt{f_{k}}}=-2\mathrm{lg}\left(\frac{\varepsilon_{k}/d_{k}}{3.76}+\frac{2.51}{Re\sqrt{f_{k}}}\right)$$

where $\varepsilon_{k}$ is the roughness of the inner surface of the pipe branch k and Re is a Reynold number of the flow in the pipe branch k.

The Newton–Raphson method is typically utilized to analyze the flow rate in each branch using the non-linear equations mentioned above. This advanced approach has been used in numerous literary works. Since the temperature of a water distribution network is relatively stable and heat loss is rare, which is different from heating a pipe network, the thermal model can be taken out of consideration. The proposed method can be used for a general network with tree-like or looped topology structures, and it is very convenient for programming. In this study, the simulation program has been developed using the Matlab platform.

## 3. Sensor Deployment Method

Two indexes are defined to evaluate the various pressure sensor combinations for leak identification in a WDN. One is *DCR*, the detection coverage rate, which ranges from zero to one and is related to false negatives. The other is *TDS*, the total detection sensitivity, linked to false positives. A larger value in the metering data makes leakage events more obvious. If the *DCRs* for several pressure sensor combinations are identical, then the *TDS* can be utilized to identify the best option. The *DCR* is calculated using Equations (12) and (13). If event *j* can be detected by a certain deployed pressure sensor within *N* sensors, this means that the event *j* is detectable, and the total number of detectable events is the $NS_{detected}$ under the corresponding *N* sensors. The identification of whether the *j-*th event $E_{j}\;$ can be detected by the *i-*th sensor $\;S_{i}$ is used to compare the pressure residual and the detection threshold at the i-th sensor node for leakage event  j. The pressure residual $\Delta P_{i,j}$ is calculated by Equation (14), which can be obtained from the simulation results. The detection threshold $\;\Delta P_{i,j}^{threshold}$ is related to the accuracy of $\;S_{i}$, and in Equation (15), the normal pressure difference $\Delta P_{i,j}^{normal}\;$ is generally assumed to be zero [26]. Therefore, $\Delta P_{i,j}^{threshold}$ is equal to half of the sensor accuracy $\delta_{sensor}$. The *TDS* is defined by Equation (16), and the quantified pressure residual is shown as the sensor deviation as follows:

(12) $$DCR=\frac{NS_{detected}}{NS_{total}}=\frac{\sum_{i=1}^{N}\sum_{j=1}^{NS_{total}}D_{i,k}}{NS_{total}}$$

where the DCR is the detection coverage rate, which ranges from zero to one, $NS_{detected}\;$ is the total number of detectable events by the set of sensors, $NS_{total}$ is the total number of pipe leakage events, i is the sensor index, and *j* is the event index, which is calculated as follows:

(13) $$D_{i,j}=\{\begin{array}{c} 1\;\;if\left(E_{j}\;is\;detected\;by\;S_{i}\;but\;not\;by\;\left\{S_{1},\;...,\;S_{i-1}\;\right\}\right)\\ 0\;\;\;\;otherwise \end{array}$$

where $E_{j}$ is the *j-*th event and $S_{i}\;$is the *i-*th sensor. If $\Delta P_{i,j}$ ≥ $\Delta P_{i,j}^{threshold}$, then $E_{j}\;\mathrm{is}\;\mathrm{detected}\;\mathrm{by}\;S_{i}\;$; otherwise, it is not detected. $\Delta P_{i,j}\;\mathrm{is}\;\mathrm{calculated}\;\mathrm{as}\;\mathrm{follows}:$

(14) $$\Delta P_{i,j}=P_{i,j}^{L}-P_{i,j}^{C}\;i=1,2,\;\ldots ,\;N,$$

where $\Delta P_{i,j}$ is the pressure residual at the *i*-th sensor node for leakage event j, $P_{i,j}^{L}$ is the pressure at the i-th sensor when leakage event *j* occurs, $P_{i,j}^{C}$ is the corresponding normal pressure at the i-th sensor under the same usage patterns of event j, and N is the number of pressure sensors. $\Delta P_{i,j}^{threshold}\;\mathrm{is}\;\mathrm{calculated}\;\mathrm{as}\;\mathrm{follows}:$

(15) $$\Delta P_{i,j}^{threshold}=1/2\delta_{sensor}+\left|\Delta P_{i,j}^{normal}\right|$$

where $\Delta P_{i,j}^{threshold}$ is the detection threshold at the i-th sensor node for leakage event j, $\delta_{sensor}\;$is the sensor accuracy, and $\Delta P_{i,j}^{normal}\;$ is the normal pressure difference at the i-th sensor node for leakage event j. The *TDS* is calculated as follows:

(16) $$TDS=\overset{N}{\underset{i=1}{\sum }}\overset{NS_{total}}{\underset{j=1}{\sum }}DS_{i,j}=\overset{N}{\underset{i=1}{\sum }}\overset{NS_{total}}{\underset{j=1}{\sum }}round\left(\frac{\Delta P_{i,j}}{\delta_{sensor}}\right)\;$$

where TDS  is the total detection sensitivity, which indicates the detection difficulty, where a larger value means that the events are more obvious on the devices, and $DS_{i,j}$ is the detection sensitivity of the $\;S_{i}$ under the leakage event $E_{j}$.

**Figure 3:**
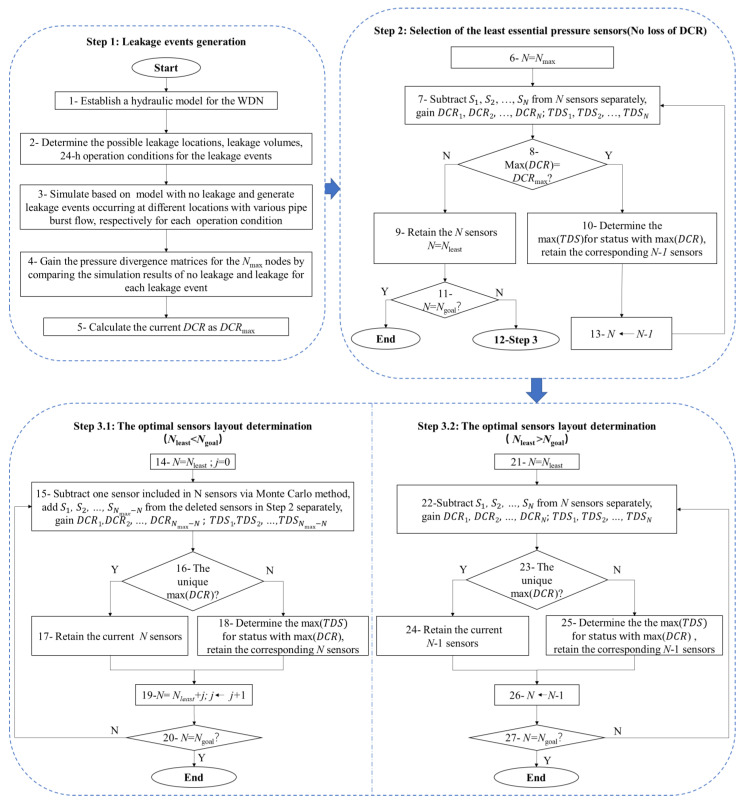
Flowchart of the proposed approach for pressure sensor deployment optimization for leak identification in WDNs.

The methodology consists of three steps, as shown in Figure 3. In step one, a number of leakage events are generated, which are essential for the leak identification analysis, and, for this, a simulation is the most convenient channel. The simulation model employed in this paper is interpreted in part two, which can be available for a burst pipe at any location. Firstly, a hydraulic model for the WDN is established. Secondly, the schemes for the leakage events are designed and specified, including the leakage locations, burst flows, boundary conditions for the sources, tanks, users, etc. Thirdly, the simulation results for each leakage scheme and the corresponding results for a scheme with no leakage are achieved, and then the pressure divergence matrices for the *N*_max_ nodes can be obtained by comparing the results for leakage and no leakage events. Finally, the *DCR* is calculated and recognized as *DCR*_max_.

In step two, the least amount and distribution of the pressure sensors are obtained through subtraction. This step is aimed at identifying the least essential pressure sensors on the promise of no reductions in the number of detection events. Beginning at the maximum value of *N*_max and_ deleting one from the current sensors $S_{1}$, $S_{2}$, …, and $S_{N\;}$, respectively, we can compare the maximum values for the *DCR* using these combinations. If the maximum *DCR* is equal to *DCR*_max_, we can determine the largest *TDS* of the sensor combinations with maximum *DCR* and retain the corresponding *N*-1 sensors. We then repeat the previous steps to delete further sensors one by one until the maximum *DCR* is less than the value of *DCR*_max_ and the lowest number and the layout of the pressure sensors are achieved.

As for step three, the compensated pressure sensors are added as allowed by the economic budget. If *N*_least_ is more than *N*_goal,_ this indicates that the detected coverage rate has to be decreased. Under these circumstances, to subtract one from the current sensors $S_{1}$, $S_{2}$, …, and $S_{N\;}$, respectively, we retain the combination with the maximum *DCR*. If there is more than one status that shares the same maximum *DCR*, we choose the combination with the maximum *TDS*. We then repeat these steps until the number of pressure sensors is decreased to *N*_goal_. If *N*_least_ is less than *N*_goal,_ this means there are extra sensors that can be installed in addition to those in the essential locations, and the extra sensors are determined based on supposing partial essential sensors are broken. By subtracting one sensor included in the essential sensors, as selected in step two via the Monte Carlo method, the *DCR* is deduced, and then we add one from the deleted sensors $S_{1}$, $S_{2}$, …, and $S_{N\max-N+1\;}$, respectively. The added sensor that results in the largest *DCR* is preserved, and if the largest *DCR* corresponds to more than one combination, the *TDS* is employed to choose the relatively optimal one. Then, supposing another sensor is broken, we select the sensor that can best take the place of the broken one. These steps are repeated until the number of the least essential sensors plus the added sensors is equal to the expected value of *N*_goal_.

## 4. Case Study

**Figure 4:**
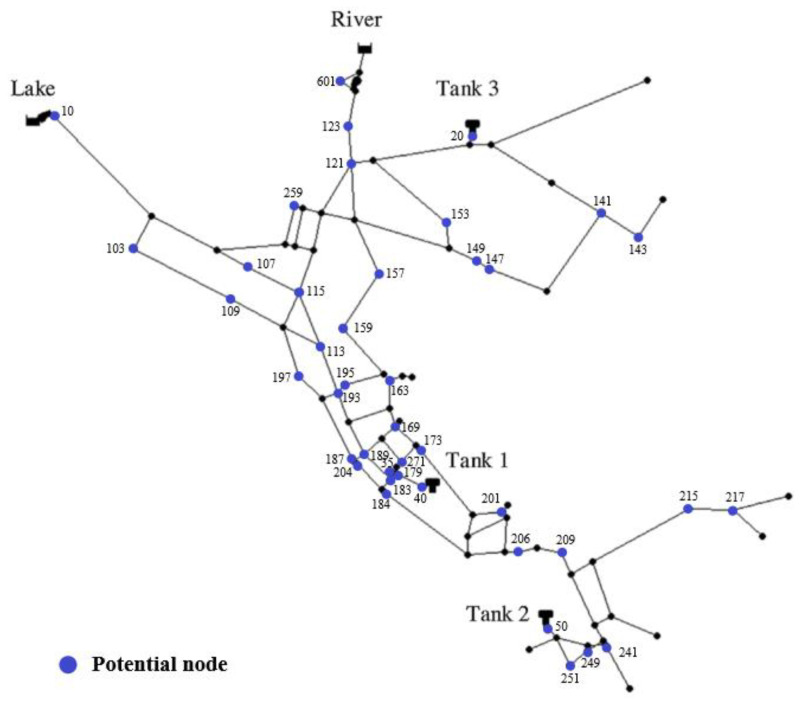
Layout of the Net3 network.

The proposed three-stage optimization decision-making method for sensor placement was applied to the well-known example network Net3, as shown in Figure 4. The specific Net3 network specification files are publicly available and included with the EPANET Programmers toolkit [34]. Many studies have been conducted on this virtual network, including those of Schwetschenau et al. (2019) [35], Mengke Zhao et al. (2020) [26], Juan Li et al. (2019) [25], and Kegong Diao et al. (2016) [36], since real-world networks are not readily available due to security issues. Our model consists of 92 junctions, 117 pipes, 2 reservoirs (1 lake and 1 river), and 3 tanks. To imitate the realistic fact that only partial nodes meet the objective requirements of sensor installation, 42 nodes are selected randomly to be powered and marked, as shown in Figure 2, as the potential locations where the pressure sensors can be installed. Different sensor thresholds may change the final results, and this paper is aimed at the method of instruction, without discussing the influence of the sensor thresholds. $\delta_{sensor}$ is regarded as 1 kpa. The number of expected sensors, *N*_goal_, is assumed to be 21, which is equal to half of the potential nodes.

### 4.1. Leakage Event Generation

Using the Net3 network, a number of leakage events are generated via simulation, and we can suppose the following: (1) all pipes are considered, and the three points that are evenly distributed in each pipeline are supposed to burst, that is, 1/4, 1/2, and 3/4 positions along the pipelines; (2) all nodes are included while the reservoirs and tanks are excluded; (3) for each leakage point, the burst flow is divided into three different statuses, that is, 1%, 3%, and 6% of the flow rate under the fault-free operating condition, respectively; (4) 24 h leakage events are generated and the hourly water demand profile is set to refer to real water bill data from a certain area of China, as shown as Table 1, where the α indicates the demand coefficient in the demand pattern. The peak demand is approximately 3000 m^3^/h at 19:00 h; and (5) the boundary condition for the reservoirs and tanks is simulated as a representative pressure for each leakage event. Therefore, the number of generated leakage events is 31896.

**Figure 5:**
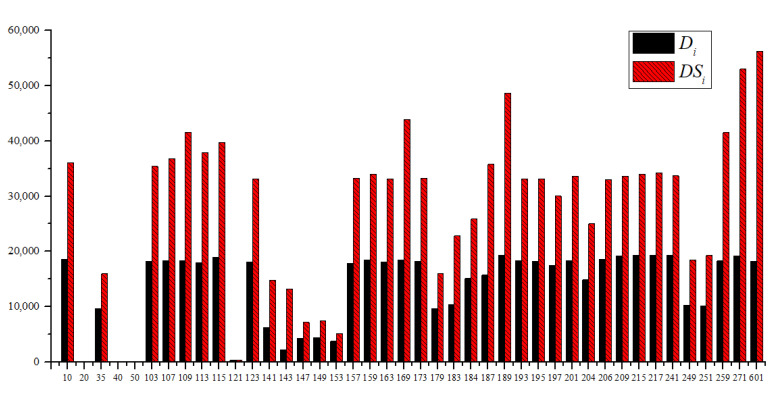
The $D_{i}$ and $DS_{i}$ values for each potential node.

The $D_{i}$ and $DS_{i}$ for each potential node are analyzed in Figure 5, indicating the leak identification ability when there is only one pressure sensor. It can be seen that the largest $D_{i}$ occurs at node 241, where the value of $D_{i}$ is 19,362 and that of the *DCR* is 60.7%, and node 601 has a superior $DS_{i}$ of 56,256. However, the values of both $D_{i}\;$ and $DS_{i}$ for nodes 20, 40, and 50 are zero, meaning that all leakage events have rare impacts on these nodes. These nodes are close to the three tanks, and the tanks are set at a classical pressure during the simulation, with the pressures for the tanks being relatively low compared to the supply pressures of the sources.

### 4.2. The Least Essential Pressure Sensor Selection

**Figure 6:**
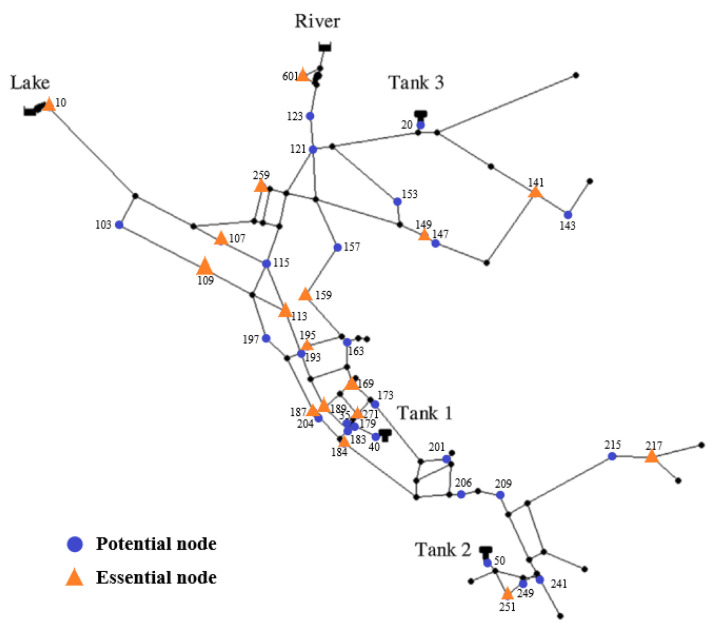
Layout of the least essential pressure sensors for the Net3 network.

In terms of the pressure sensor deployment for the 42 potential nodes, the *DCR*_max_ is 91.35%, and none of these 42 sensors can detect 8.65% of the leakage events, mainly due to the tiny leakage volumes and marginal leak locations. Beginning at the maximum number of sensors, one sensor is removed from the forty-two sensors at a time and the results show that when the sensor installed in node 50 is removed, the *DCR* is identical to the *DCR*_max_ and the *TDS* is the maximum value for this status for the *DCR*_max_. Node 50 is specified to be subtracted, and then one sensor is removed from the remaining forty-one sensors at a time and the previous steps are repeated. The process is listed in Table 2, and it is obvious that when the number of sensors decreased from 17 to 16, the optimal *DCR* for the 16 sensors is less than 91.35%, indicating that some leakage events were not detected. Therefore, the minimum number of essential pressure sensors, *N*_least_, is 17, and the pressure sensor layout is a combination of nodes 10, 107, 109, 113, 141, 149, 159, 169, 184, 187, 189, 195, 217, 251, 259, 271, and 601, as shown in Figure 6.

### 4.3. The Optimal Sensor Layout Determination

**Figure 7:**
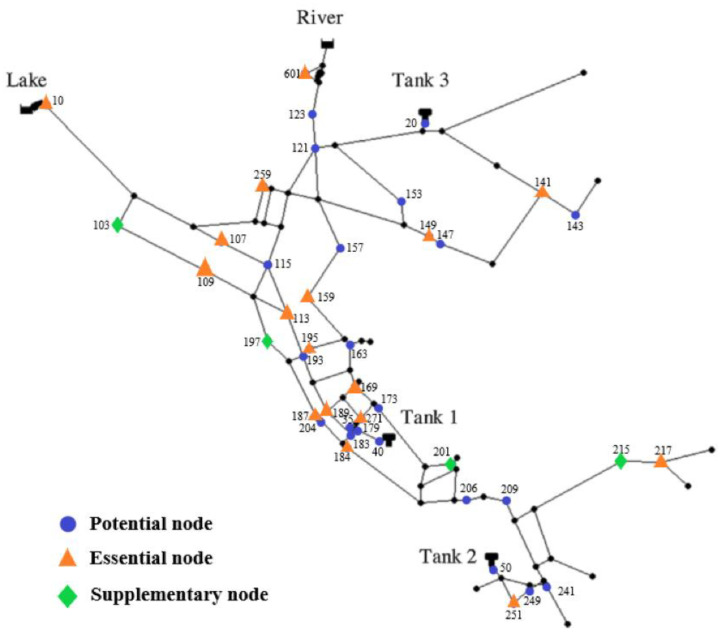
Layout of the final pressure sensors for the Net3 network.

The number of the least essential pressure sensors is 17, which is 4 less than the expected 21. There are various faults that can occur with the sensors, such as becoming stuck, damaged, etc., which leads to instrument failure. The extra four sensors are expected to take the place of the failed sensors. The process in this section is exactly as shown in Table 3. Firstly, the Monte Carlo method is utilized and the sensor located in node 217 is supposed to have failed, which is obvious as the *DCR* declines by 2.94% and some leakage events cannot be detected. Then, one of the twenty-five sensors removed in step two is sequentially added, and node 215 is identified to have the pressure sensor installed for the best compensatory performance with a *DCR* of 91.35%. We repeat these steps and the sensor located in node 189 is removed, and nodes 197, 204, and 183 share the same *DCR* whether or not one of them takes the place of node 189. Then, node 197 is left due to having the highest *TDS* value. Similarly, node 201 replaces node 271 and node 103 replaces node 10. Finally, the extra sensors added in this step are located in nodes 215, 197, 201, and 103. Hence, the optimal 21 sensors are located in nodes 10, 107, 109, 113, 141, 149, 159, 169, 184, 187, 189, 195, 217, 251, 259, 271, 601, 215, 197, 201, and 103, respectively, as shown as Figure 7.

To evaluate the performance of the ultimate determined sensor layout, a set of test data is generated by simulation, with no involvement in the sensor selection process. The boundary conditions, used to obtain the leakage samples in step one, should reflect the design scenario or typical operating condition. This ensures congruity between the load distribution of the samples and the prevailing empirical distribution patterns. Therefore, the water load of each user experiences random fluctuations within a range of ±10% based on the training samples, while keeping the other settings and topology of WDNs unaltered, generating new 24 h leakage events for testing. Applying the final 21 pressure sensors to the test data, it is found that the *DCR* of the final sensor topology is 92.03% while the *DCR*_max_ of 42 points stands at 92.05%. The *TDS* of the final sensor topology is calculated as 786,973 while the *TDS* of 42 points is 1,176,820. The result shows that this sensor layout exhibits a commendable leak monitoring efficacy, particularly when confronted with analogous water consumption patterns.

### 4.4. Impact of Leakage Model Parameter on Results

The previous section presented the application steps of the proposed method, leading to the final optimized layout. The generation of leakage samples during the initial step holds implications for the resultant outcomes. Ideally, a sample containing all possible leak locations and leak flow rates would be considered comprehensive and advantageous. However, due to computational power and time limitations, it is impracticable to comprehensively encompass all possible leakage events. Therefore, the influence of leak model parameter settings on the results is discussed in this section, to provide a reference for the sample generation process.

In Section 4.1, the leakage locations are configured to encompass all nodes, as well as 1/4, 1/2, and 3/4 positions along the pipelines. The burst flow is divided into three distinct levels, namely 1%, 3%, and 6% of the flow rate calculated under fault-free operating conditions. These samples were individually selected to derive 21 optimized sensor layouts using the proposed method in this paper. Subsequently, these resulting sensor layouts are applied to the entire sample set and their corresponding *DCR* and *TDS* are determined.

The specific outcomes are shown in Table 4, with nodes arranged in reverse order of $D_{i}$.

It can be seen from the table that the optimal sensor points obtained from these seven leakage samples are relatively concentrated. There are sixteen nodes, such as nodes 217, 189, 271, etc., chosen to be the final point by all seven cases. It indicates that 16 out of 21 points are identical among these results and the other 5 points are distributed among ten nodes, such as nodes 215, 241, 209, etc. The leak identification abilities in the results gained by the sensor deployment method proposed are not bad with the worst *DCR* at 91.11%, which means losing the ability to detect 77 leakage events. The *DCRs* pertaining to different leak locations exhibit relative similarity and are close to the maximum *DCR* compared to the *DCRs* gained from three statuses of burst flow. This can be attributed to the limited influence of leakage positions among the identical pipelines on the pressure distribution, particularly when the pipeline is not long, compared to the magnitude of the leakage. The *TDSs* gained by separated burst flow samples are larger than others with the chosen nodes show decreased *DCRs*in combination. In general, results based on a set of more comprehensive leakage samples exhibit an improved ability to reflect leakage events. Given limited computing power and time constraints, the leakage location can be set simply while placing greater emphasis on the burst flow setting. It is suggested to generate a comprehensive range of leakage events with detailed consideration of varying possible magnitudes of leakage.

## 5. Conclusions

This paper is aimed at pressure sensor deployment optimization in terms of leak identification in WDNs, and a methodology is proposed to achieve the best leakage fault detection performance given several uncertain restrictions. The methodology is made up of three steps: firstly, a hydraulic model is established and a number of leakage events are generated. Secondly, the least essential pressure sensors are selected by subtracting the sensors sequentially from all the potential installed nodes, with no losses in the detected events. Lastly, if the smallest number is less than expected, we suppose a certain sensor has failed and identify another location to best take its place among the removed sensors from the previous step, and this is repeated until the sensor number is up to the expected number and the optimal deployment is achieved. Otherwise, we subtract the sensors with the optimal *DCR* values from the expected number. A Net3 network is employed as a case study to demonstrate the specific process of the proposed methodology, and the results show that the methodology is convenient and practical for leak identification in WDNs. The main conclusions are summarized as follows:

(1) A novel methodology is proposed in this paper to optimize the deployment of pressure sensors for leak identification in WDNs. The methodology considers economic budgets, sensor faults, and the available local conditions for installation, which are proper and practical elements for realistic projects. The economic budgets are associated with the number and accuracy of the sensors, and both of these are considered in the methodology and influence the results.

(2) Two indexes are defined to evaluate the effectiveness of sensor layout for leak identification. One is *DCR*, related to false negatives, and the larger the value, the more leakage events can be reflected in the measured values. The other is *TDS*, linked to false positives, and the larger the value, the more obvious it is to detect the leakage events. The principle is to determine priority to ensure an optimal *DCR* and retain the largest *TDS* with an identical *DCR.*

(3) The lowest number of pressure sensors can be obtained via the proposed approach without leakage event detection loss, and it can be beneficial for planning developments and avoiding wasted manpower and money. Otherwise, if the budget allows, the final deployment can be obtained via a Monte Carlo method by adding the compensated sensors based on the hypothesis that there has been a partial sensor failure.

(4) A well-known WDN is employed to illustrate the methodology in detail, and up to 91.35% of leakage events can be detected under the restriction of the installation locations for sensors. The results show that at least 17 pressure sensors are required to guarantee the detection of leaks, and the sensitivity of the *TDS* is approximately half of the 42 locations installed. For the optimal number of sensors (twenty-one) with a budget, the *DCR* can be 90.83%, and the *TDS* is slightly decreased when four sensors fail and another four locations are replaced.

## Figures and Tables

**Table 1:** Hourly water demand load rates.

Hour	α	Hour	A	Hour	α	Hour	α
1	0.0865	7	0.9312	13	0.3230	19	1.0000
2	0.0661	8	0.8195	14	0.2314	20	0.8984
3	0.0350	9	0.7670	15	0.2200	21	0.8242
4	0.0338	10	0.7626	16	0.2715	22	0.3031
5	0.1631	11	0.8355	17	0.7152	23	0.1333
6	0.7099	12	0.7764	18	0.8978	24	0.1044

**Table 2:** The process of the least essential pressure sensor selection for the Net3 network.

Number of Sensors	Optimal *DCR* (%)	Optimal *TDS*	Removed Location	Number of Sensors	Optimal *DCR* (%)	Optimal *TDS*	Removed Location
41	91.35	1,163,630	50	28	91.35	976,387	206
40	91.35	1,163,630	40	27	91.35	943,294	123
39	91.35	1,163,630	20	26	91.35	910,197	193
38	91.35	1,163,311	121	25	91.35	877,095	163
37	91.35	1,158,150	153	24	91.35	843,807	173
36	91.35	1,150,979	147	23	91.35	810,495	157
35	91.35	1,137,732	143	22	91.35	776,897	209
34	91.35	1,121,772	35	21	91.35	743,264	201
33	91.35	1,105,780	179	20	91.35	709,547	241
32	91.35	1,087,334	249	19	91.35	675,538	215
31	91.35	1,064,518	183	18	91.35	640,128	103
30	91.35	1,039,493	204	17	91.35	600,396	115
29	91.35	1,009,458	197	16	91.34	556,527	169

**Table 3:** The process of the optimal sensor layout determination for the Net3 network.

Item		Current Pressure Sensor Deployment																	Indexes	
																			*DCR*	*TDS*
First	I	10	107	109	113	141	149	159	169	184	187	189	195	217	251	259	271	601	91.35%	600,396
	II	10	107	109	113	141	149	159	169	184	187	189	195		251	259	271	601	88.41%	564,986
	III	10	107	109	113	141	149	159	169	184	187	189	195	215	251	259	271	601	91.35%	598,995
Second	I	10	107	109	113	141	149	159	169	184	187	189	195	215	251	259	271	601	91.35%	598,995
	II	10	107	109	113	141	149	159	169	184	187		195	215	251	259	271	601	91.34%	550,310
	III	10	107	109	113	141	149	159	169	184	187	197	195	215	251	259	271	601	91.35%	580,345
		10	107	109	113	141	149	159	169	184	187	204	195	215	251	259	271	601	91.35%	575,335
		10	107	109	113	141	149	159	169	184	187	183	195	215	251	259	271	601	91.35%	573,126
Third	I	10	107	109	113	141	149	159	169	184	187	197	195	215	251	259	271	601	91.35%	580,345
	II	10	107	109	113	141	149	159	169	184	187	197	195	215	251	259		601	90.64%	527,327
	III	10	107	109	113	141	149	159	169	184	187	197	195	215	251	259	183	601	90.83%	550,143
		10	107	109	113	141	149	159	169	184	187	197	195	215	251	259	201	601	90.83%	560,960
Fourth	I	10	107	109	113	141	149	159	169	184	187	197	195	215	251	259	201	601	90.83%	560,960
	II		107	109	113	141	149	159	169	184	187	197	195	215	251	259	201	601	90.81%	524,856
	III	103	107	109	113	141	149	159	169	184	187	197	195	215	251	259	201	601	90.83%	560,266

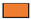
 represents the hypothetical failed sensors and 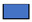 represents the replacement supplementary sensors.

**Table 4:** Optimal sensor layout obtained from different leakage samples.

Node	Total Samples	Leakage Location			Burst Flow		
		Loc_0.25	Loc_0.5	Loc_0.75	Flow_1%	Flow_3%	Flow_6%
217	○	○	○	○	○	○	○
215	△	-	-	-	-	-	△
241	-	-	-	△	△	△	△
189	○	△	○	○	△	○	○
271	○	○	○	○	○	○	○
209	-	○	○	-	-	-	○
115	-	△	○	-	○	-	△
10	○	-	-	○	○	○	-
206	-	-	-	△	△	△	-
159	○	○	○	○	○	○	○
193	-	-	-	-	-	-	-
201	△	△	△	○	○	△	△
109	○	○	○	△	△	○	○
169	○	○	△	○	△	○	○
173	-	-	-	-	-	-	-
107	○	○	○	○	○	○	○
103	△	○	△	△	○	△	△
195	○	○	○	○	○	○	○
123	-	-	-	-	-	-	-
259	○	○	○	○	○	○	○
163	-	-	-	-	○	○	-
601	○	○	○	○	○	○	○
113	○	○	○	○	○	○	○
157	-	-	-	-	-	-	-
197	△	△	△	△	△	△	△
187	○	△	○	-	-	○	-
184	○	○	○	○	○	○	○
204	-	-	-	-	-	-	-
183	-	-	-	-	-	-	-
249	-	-	-	-	-	-	-
251	○	○	○	○	-	-	○
35	-	-	-	-	-	-	-
179	-	-	-	-	-	-	-
141	○	○	○	○	-	-	-
149	○	○	○	○	○	○	○
147	-	-	-	-	-	-	-
153	-	-	-	-	-	-	-
143	-	-	-	-	-	-	-
121	-	-	-	-	-	-	-
20	-	-	-	-	-	-	-
40	-	-	-	-	-	-	-
50	-	-	-	-	-	-	-
*DCR*	91.35%	91.31%	91.31%	91.32%	91.23%	91.24%	91.11%
*TDS*	733,483	736,700	736,700	730,479	769,253	765,304	753,873

○ represents the essential sensors and △ represents the supplementary sensors.

## Data Availability

The data presented in this study are available on request from the corresponding author.
